# Notes for authors

**DOI:** 10.1155/S1463924686000342

**Published:** 1986


column derivatization for amino-acid analysis. It has proved to be well-suited to the analysis of foods.

### Acknowledgement

This work forms part of Project M48, 'Carbohydrate—protein interactions during the thermal processing of foods', funded by the Ministry of Agriculture, Fisheries and Foods.

### References

1. Heinrikson, R. L. and Meredith, S. C., *Analytical Biochemistry*, **136** (1984), 65.

2. Bidlingmeyer, B. A., Cohen, S. A. and Tarvin, T. L., *Journal of Chromatography*, **336** (1984), 93.

3. Moore, S., Spackman, D. H. and Stein, W. H., *Analytical Chemistry*, **30** (1958), 1185.

4. Gordon, W. G. and Whittier, E. O., *Fundamentals of Dairy Chemistry*, Ed. Webb, B. H. and Johnson, A. H. (The AVI Publishing Co, 1965), 60.

### ENDOWMENT FUND CREATED TO HONOR THE LATE TOMAS HIRSCHFELD

In memory of Tomas Hirschfeld, and his contributions to applied chemistry, the Center for Process Analytical Chemistry at the University of Washington has created a special endowment fund. In addition to Tomas's position as Senior Staff Scientist at Lawrence Livermore National Laboratory, he was a CPAC researcher and an Affiliate Professor in the Department of Chemistry at the University of Washington.

Contributions to the fund are being handled by the Gift Processing Office at the University of Washington, AJ-55, Seattle, WA 98195. According to Deborah Illman, Assistant Director of CPAC, one of the things the endowment will support is a special graduate student fellowship at CPAC.

Hirschfeld will be remembered for his contributions to the fields of spectroscopy, fibre optics and their use in process analytical chemistry. Evidence of the importance of these contributions comes from the numerous awards he received in his 25-year career including: the Meggar's Award (Society for Applied Spectroscopy, 1978); the Louis A. Strait Award (Society for Applied Spectroscopy, 1984); the Pittsburgh Spectroscopy Award (1986); IR-100 Awards (1975, 1977, 1981, 1983, 1985).

### NOTES FOR AUTHORS

*Journal of Automatic Chemistry incorporating Journal of Clinical Laboratory Automation* covers all aspects of automation and mechanization in analytical, clinical and industrial environments. The *Journal* publishes original research papers; short communications on innovations, techniques and instrumentation, or current research in progress; reports on recent commercial developments; and meeting reports, book reviews and information on forthcoming events. All research papers are refereed.

### Manuscripts

Two copies of articles should be submitted. All articles should be typed in double spacing with ample margins, on one side of the paper only. The following items should be sent: (1) a title-page including a brief and informative title, avoiding the word 'new' and its synonyms; a full list of authors with their affiliations and full addresses; (2) an abstract of about 250 words; (3) the main text; (4) appendices (if any); (5) references; (6) tables, each table on a separate sheet and accompanied by a caption; (7) illustrations (diagrams, drawings and photographs) numbered in a single sequence from 1 upwards and with the author's name on the back of every illustration; captions to illustrations should be typed on a separate sheet. Papers are accepted for publication on condition that they have been submitted only to this *Journal*.

### References

References should be indicated in the text by numbers following the author's name, i.e. Skeggs [6]. In the reference section they should be arranged thus:

to a journal

Manks, D. P., *Journal of Automatic Chemistry*, **3** (1981), 119.

to a book

Malmstadt, H. V., in *Topics in Automatic Chemistry*, Ed. Stockwell, P. B. and Foreman, J. K. (Horwood, Chichester, 1978), p. 68.

### Illustrations

Original copies of diagrams and drawings should be supplied, and should be drawn to be suitable for reduction to the page or column width of the *Journal*, i.e. to 85 mm or 179 mm, with special attention to lettering size. Photographs may be sent as glossy prints or as negatives.

### Proofs and offprints

The principal or corresponding author will be sent proofs for checking and will receive 50 offprints free of charge. Additional offprints may be ordered on a form which accompanies the proofs.

*Manuscripts should be sent to either Dr P. B. Stockwell or Ms M. R. Stewart, see inside front cover.*

